# Randomized, Multicenter, Double–Blind Study of the Safety and Efficacy of 1%D-3-Hydroxybutyrate eye drops for Dry Eye Disease

**DOI:** 10.1038/srep20855

**Published:** 2016-02-11

**Authors:** Tetsuya Kawakita, Miki Uchino, Kazumi Fukagawa, Kenichi Yoshino, Seika Shimazaki, Ikuko Toda, Mari Tanaka, Hiroyuki Arai, Keiko Sakatani, Seiichiro Hata, Takashi Okano, Kazuo Tsubota

**Affiliations:** 1 Department of Ophthalmology, Keio University School of Medicine, 35 Shinanomachi, Shinjuku, Tokyo Japan; 2 Ryogoku Eye Clinic, 4-33-12, Ryogoku, Sumida, Tokyo Japan; 3 Iidabashi Eye Clinic, 3-10-10, Iidabashi, Chiyoda, Tokyo Japan; 4 Yoshino Eye Clinic, 1-20-10, Ueno, Taito, Tokyo Japan; 5 Shimazaki Eye Clinic, 2—4—13, Nagatacho, Chiyoda, Tokyo Japan; 6 Minamiaoyama Eye Clinic, 3-3-11, Kitaaoyama, Minato, Tokyo Japan; 7 Yatsu Ekimae Azisai Eye Clinic, 4—6—19, Yatsu, Narashino, Chiba, Japan; 8 Minatomirai Eye Clinic, 4-7-1, Minatomirai, Nishi, Yokohama Kanagawa Japan; 9 Sky building Eye Clinic, 2-19-12, Takashima, Nishi, Yokohama, Kanagawa Japan; 10 Smile Eye Clinic, 1-6-12, Aobadai, Aoba, Yokohama, Kanagawa Japan

## Abstract

In a previous study, we demonstrated that topical D-*beta*-hydroxybutyrate ameliorates corneal epithelial erosion and superficial punctate keratopathy in a rat model of dry eye disease. In the current investigation, we performed a prospective, randomized, multicentre, double-blind, placebo-controlled study to assess the safety and efficacy of 1% D-3-hydroxybutyrate eye drops in patients with dry eye disease. A total of 65 patients were randomly assigned to either the placebo group or the 1% D-3-hydroxybutyrate group, and the treatments were administered 6 times a day for 4 weeks. We then evaluated corneal fluorescein staining, corneal and conjunctival rose Bengal staining, tear film break-up time (BUT), Schirmer score, and subjective symptoms. At both 2 and 4 weeks, the corneal rose Bengal score was significantly better in the 1% D-3-hydroxybutyrate group than in the placebo group. Among patients with an initial Schirmer score of ≤5 mm, the corneal fluorescein staining score was significantly better in the 1% D-3-hydroxybutyrate group than in the placebo group at two weeks. Mild ocular symptoms occurred in both groups, and these spontaneously resolved. The present study suggested that 1% D-3-hydroxybutyrate eye drops are safe and effective in treating ocular surface disorders in patients with tear-deficient dry eye disease.

Dry eye disease is characterised by chronic tear deficiency that has a multifactorial aetiology^1^. A population-based assessment estimated that dry eye disease occurs in 10% to 20% of the adult population. Using dry eye disease therapy, clinicians aim to recover endogenous tear function, and thereby restore a proper environment to the ocular surface. Dry eye is commonly treated by frequently applying a viscoelastic artificial tear solution, by wearing moisture chamber spectacles, or by lacrimal punctal occlusion, which can involve either surgery or plug insertion^2^. However, these treatments only preserve tear fluid to prevent dehydration of the ocular surface. To improve tear retention and increase tear stability on the ocular surface, diquafosol sodium (DIQUAS^®^; Santen Pharmaceutical Ltd.) and rebamipide (Mucosta^®^; Otsuka Pharmaceutical Ltd.) are currently approved as medical eye drops in Japan. However, the only available pharmacologically active eye drop for dry eye disease is cyclosporine A (Restasis^®^; Allergan Inc., Irvine, CA), which has an immunological effect involving inflammation in the conjunctiva^3^,^4^,^5^. Many investigators have effectively managed dry eye disease using autologous serum, the composition of which resembles tears^6^. Moreover, this approach has been used in dry eye disease of many different causes—chronic graft-versus-host disease^7^, persistent epithelial defects, vital staining of the ocular surface following laser-assisted *in situ* keratomileusis^8^, and Sjögren syndrome^9^. These successful clinical results suggest that active agents, which restore homeostasis in ocular surface cells are essential components in eye drop formulations to treat dry eye disease.

D-*beta*-hydroxybutyrate (BHB) is the most abundantly produced physiological ketone. A fatty acid-derived substrate that enters the Krebs cycle as acetyl CoA, BHB is normally present at very low concentrations in human blood (0.05 mM); nonetheless, it plays a key role in mammalian energy metabolism^10^. In addition to its well-known role as an alternative energy source during glucose starvation, there is a growing body of evidence to suggest that BHB has neuroprotective effects^11^. For example, Tieu *et al*. demonstrated that BHB protects dopaminergic neurons in a mouse model of Parkinson’s disease^12^. Furthermore, cell culture studies have shown that it protects neurons in models of Parkinson’s and Alzheimer’s diseases, as well as in hypoxia^13^,^14^. More recently, it was discovered that calorie restriction spurs the production of BHB, which in turn blocks the activity of a class of enzymes called histone deacetylases and thereby helps cells resist oxidative stress. These findings indicate that BHB is therapeutically beneficial in neurological disorders. Concerning ocular surface disorders, we have shown that topical BHB ameliorates corneal epithelial erosion and superficial punctate keratopathy—both hallmarks of dry eye disease—in a rat model^15^,^16^. Our findings indicated that BHB eye drops preserve both homeostasis of the ocular surface and natural tears under dry eye conditions. The present study was designed to investigate the efficacy, safety, and patient tolerance of a BHB solution to treat ocular surface disorders in patients with dry eye disease. In a rat model of the same disease, the corneal staining fluorescence score was better in the BHB group than in the placebo group; moreover, the effect was dose-dependent (0.2–1%). Because pharmacological, toxicological, and pharmacokinetic study has confirmed the safety of 1% BHB, this concentration was selected for this study. We also performed a subgroup analysis to investigate the relationship between initial dry eye severity and the effect of the BHB ophthalmic solution.

## Results

### Participants and follow-up

A total of 77 patients were screened for inclusion in this study; ultimately, 65 were randomly assigned to one of two groups: 33 patients received the placebo, and 32 received 1% BHB eye drops. Five patients were disqualified due to protocol violations, and three were withdrawn because of adverse events—one from the placebo group, and a further two from the 1% BHB group.

### Patient demographics

The demographic characteristics of the patients are shown in Table 1; most were elderly women.

### Efficacy analysis

#### Ocular surface staining score

**Figure 1:**
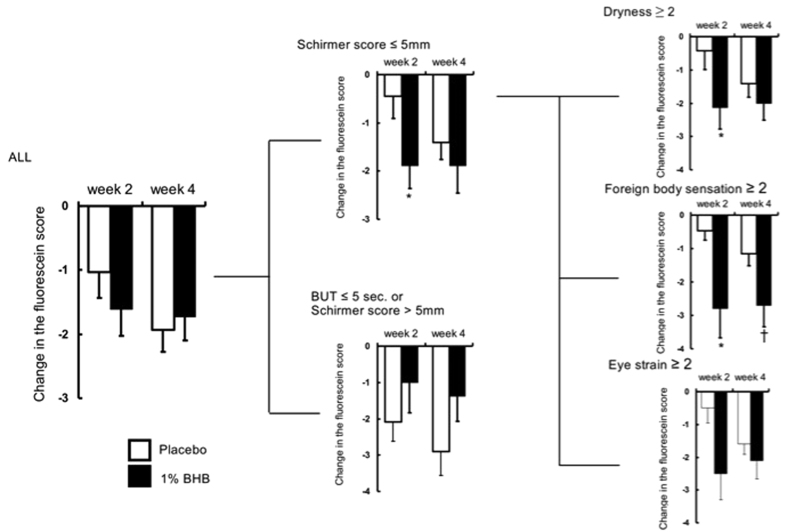
Changes from baseline in the corneal fluorescein staining score. Separate subgroup analyses were conducted depending on a Schirmer score ≤5 mm with subjective symptom score ≥2. Mean ± standard error. *P < 0.05, ^†^*P* < 0.1 significantly different from the placebo group.

**Figure 2:**
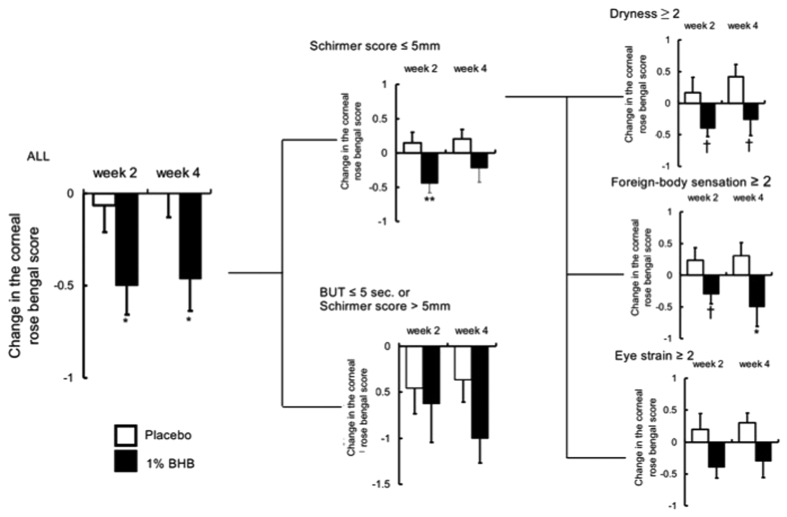
Changes from baseline in the corneal rosebengal staining score. Separate subgroup analyses were conducted depending on a Schirmer score ≤5 mm with subjective symptom score ≥2. Mean ± standard error. **P < 0.01, *P* < 0.05, ^†^*P* < 0.1 significantly different from the placebo group.

**Figure 3:**
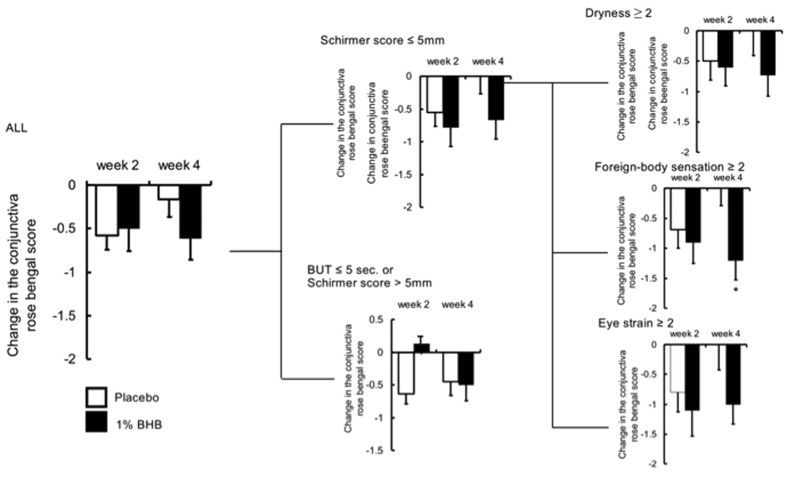
Changes from baseline in conjunctiva rose bengal staining score. Separate subgroup analyses were conducted depending on Schirmer score ≤5 mm with subjective symptom score ≥2. Mean ± standard error. *P < 0.05, ^†^P < 0.1 significantly different from the placebo group.

At both two and four weeks after the beginning of treatment, no significant differences were observed between the placebo and 1% BHB groups in terms of the corneal fluorescein staining score (Fig. 1). The corneal rose bengal score was significantly better in the 1% BHB group than in the placebo group at both two and four weeks after the beginning of treatment (p < 0.05 in both cases; Fig. 2). However, at neither time point was there a significant difference between the groups in terms of the conjunctival rose bengal staining score (Fig. 3).

#### Tear film status

**Figure 4:**
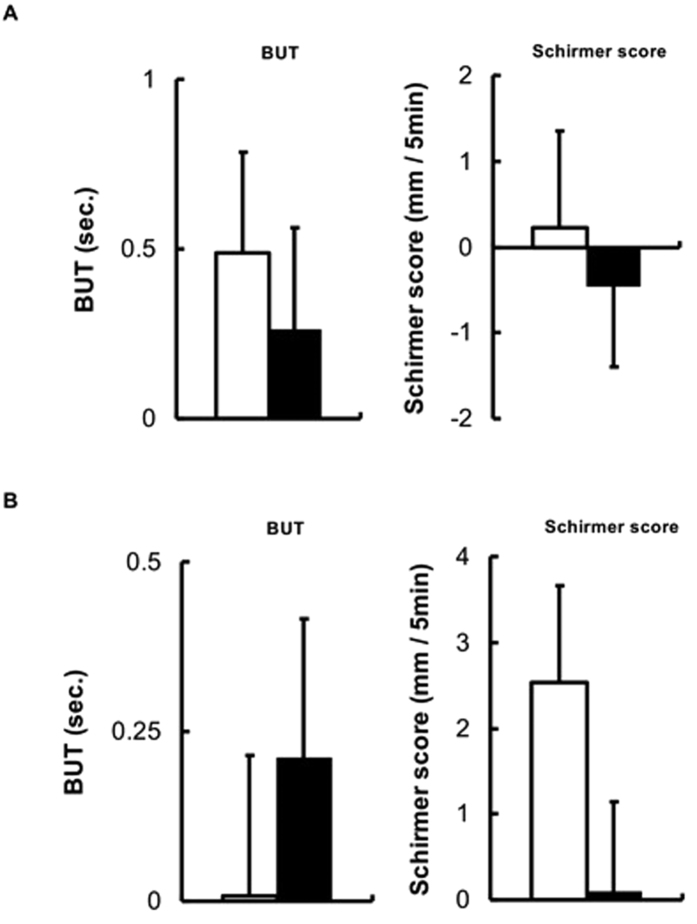
Changes from baseline in tear function. All patients (**A**), Schirmer score ≤5 mm with Foreign body sensation ≥2 group (**B**), □placebo, ■1% BHB.

No significant differences occurred between the groups at either time point in terms of either the BUT or Schirmer score (Fig. 4).

#### Subjective symptoms

**Figure 5:**
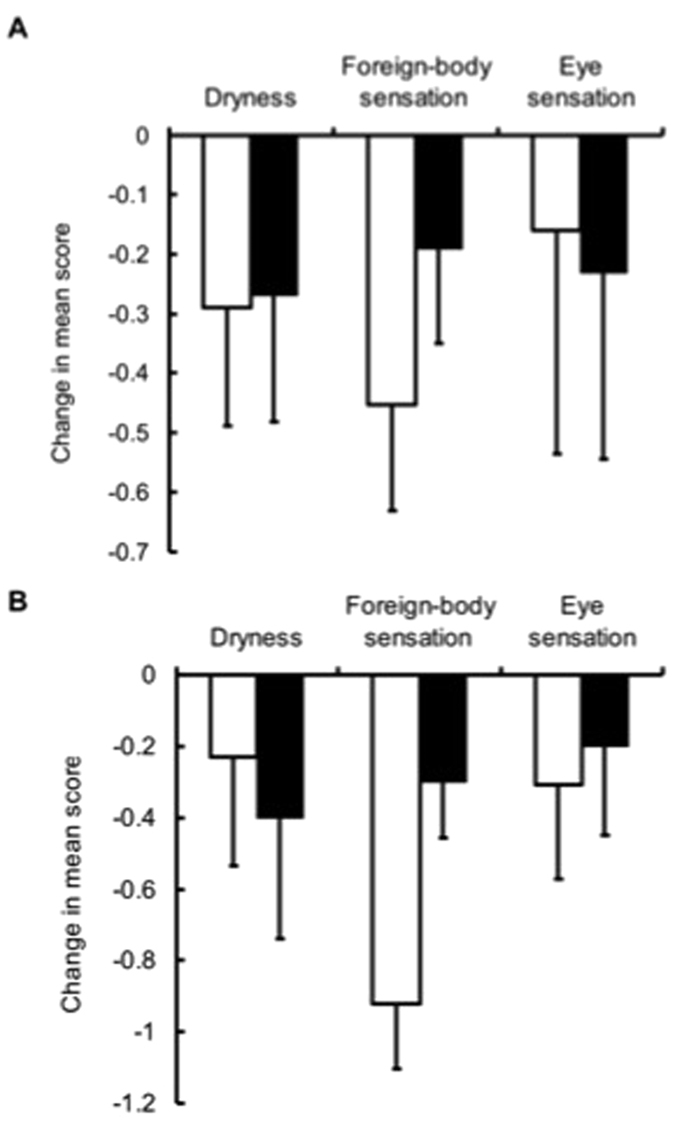
Changes from baseline in subjective symptoms. All patient (**A**), Schirmer score ≤5 mm with Foreign-body sensation ≥2 group (**B**), □placebo, ■1% BHB.

There were no significant differences between the two groups with regard to dryness, foreign body sensation, or ocular fatigue (Fig. 5).

### Subgroup analysis

**Figure 6:**
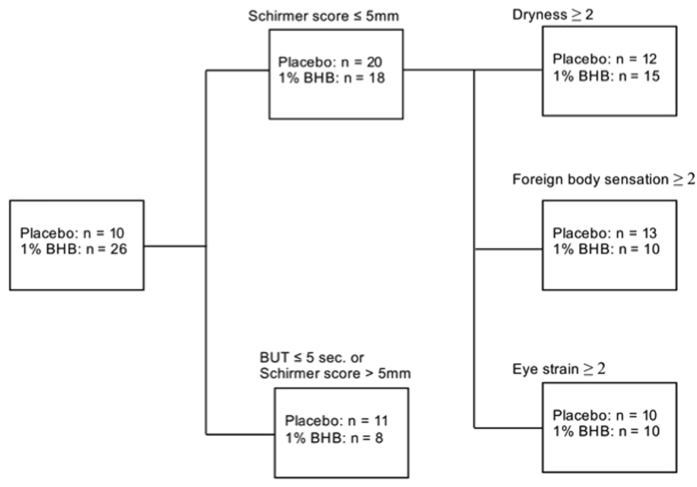
Diagram of the subgroup analysis.

The severity of dry eye was determined using the corneal staining degree, tear film status, and subjective symptoms. With regard to tear film status, the criteria for patient selection were a Schirmer score of ≤5 mm, or a BUT of ≤5 seconds. To investigate the relationship between tear secretion capacity and the effects of 1% BHB, a separate analysis was performed on a subgroup of patients: those who had an initial Schirmer score of ≤5 mm (Fig. 6). In addition, to clarify the relationship between combinations of subjective symptoms, additional subgroup analyses were performed based on each subjective symptom.

The presence of objective symptoms, or two or more subjective symptoms, was regarded as a threshold value. In the Schirmer score ≤5 mm subgroup, the corneal fluorescein staining score two weeks after the beginning of treatment was significantly better in the 1% BHB group than in the placebo group. In contrast, no significant differences were observed between the treatment groups in the Schirmer score >5 mm subgroup.

In the Schirmer score ≤5 and dryness or ocular fatigue ≥2 subgroup, the corneal fluorescein staining score two weeks after the beginning of treatment was significantly better in the 1% BHB group than in the placebo group (p < 0.05; Fig. 1). In the same subgroup, the corneal rose bengal staining score, both two and four weeks after the beginning of treatment, was slightly better in the 1% BHB group than in the placebo group.

In the Schirmer ≤5 and foreign sensation ≥2 subgroup, both the corneal fluorescein staining score and corneal rose bengal staining score two weeks after the beginning of treatment were significantly better in the 1% BHB group than in the placebo group (p < 0.05 in both cases; Fig. 2). Furthermore, the conjunctival rose bengal staining score four weeks after the beginning of treatment was significantly better in the 1% BHB group (p < 0.05; Fig. 3). In the same subgroup, both the corneal fluorescence staining score four weeks after the beginning of treatment and the corneal rose bengal staining score two weeks after the beginning of treatment were slightly better in the 1% BHB group than in the placebo group.

### Safety analysis

The safety results are presented in Table 1. Three adverse events were observed in three subjects in placebo group, while seven adverse events occurred in three subjects in the 1% BHB group. The two treatment groups did not differ significantly in terms of the incidence of adverse effects. All the adverse effects noted were mild ocular symptoms that spontaneously resolved.

## Discussion

The most important finding of this study was that treatment with the 1% BHB ophthalmic solution significantly improved both corneal and conjunctival symptoms in patients with an impaired capacity for tear secretion.

The BUT is a direct test of precorneal tear film stability, constituting the time taken for the tear film to evaporate from the exposed corneal surface^17^. The Schirmer test is used to determine whether the lacrimal gland can produce enough tears^18^. The volume or turnover rate of the tears, which supply nutrition to the cells of the ocular surface, is largely determined by the capacity for tear secretion. In the present study, treatment using 1% BHB significantly ameliorated the symptoms of ocular surface disorders in patients with decreased tear production, but not in those with normal tear secretion and BUT shortening. In addition, no significant differences were observed between the 1% BHB and placebo groups in terms of the capacity for tear secretion from the first visit to four weeks after the beginning of treatment.

A preliminary investigation in an animal model revealed that 1% BHB does not affect tear dynamics, and may rather directly reverse epithelial cell degeneration. This would lead to the normalisation of corneal epithelial differentiation under dry eye conditions^16^. Tears are secreted from the lacrimal glands, and hypofunction in this organ causes cell damage to the ocular surface, which aggravates dry eye^19^. To the best of our knowledge, there are no treatments available that can restore lacrimal function once it has been damaged. Therefore, although 1% BHB does not restore the capacity for tear secretion, it can complement the process by providing an appropriate environment for ocular surface cells.

The ocular surface epithelial staining score is correlated with the BUT. In the present study, the symptoms of ocular surface disorders improved after BHB application; however, tear film stability was not concomitantly restored. These results demonstrate that BHB has a beneficial effect on both corneal and conjunctival disorders in patients with a decreased capacity for tear secretion, but that the treatment does not improve this capacity.

Three subjective symptoms—dryness, eye strain, and foreign body sensation—were selected to assess the severity of dry eye in the present study. Ocular dryness is predominantly due to either an aqueous deficiency or excessive evaporation of the tear film; the symptom is readily affected by environmental factors such as humidity or air flow^20^. Eye strain refers to ocular fatigue, and is one of the main symptoms of dry eye^21^. Eye strain is assumed to be caused by both physiological and external factors, the latter of which includes poor accommodation, mismatches between the images in the left and right eyes, or poor lightning conditions^22^,^23^. Foreign body sensation—also known as gritty eye, sandy eye, or scratchy eye—is a more specific symptom; it occurs when the corneal epithelium breaks, thereby exposing the sensitive corneal nerves to the ocular surface^24^,^25^,^26^. In the present study, 1% BHB improved corneal and conjunctival staining more in patients with foreign body sensation than in those with dryness or eye strain. These results indicate that 1% BHB has a beneficial effect on serious corneoconjuctival epitheliopathy, a disease that often accompanies subjective symptoms.

Dry eye has been classified into two main categories: aqueous-deficient and evaporative^27^. Tear-deficient dry eye is primarily characterized by the lack of tear secretion from the lacrimal glands, while evaporative dry eye involves excessive evaporative loss of tears from the ocular surface, leading to tear film instability with normal tear secretion. This study demonstrated that tear-deficient dry eye, among the ocular surface disorders associated with dry eye syndrome, may be a target for the 1% BHB ophthalmic solution.

In the present study, a small number of patients were included, and they were evaluated for a relatively short period of time. In addition, a direct correlation between the drug mechanism of action and expected results could have been drawn more clearly. Further studies are needed to clarify the effects and safety of prolonged application of BHB ophthalmic solution at different concentrations.

## Materials and Methods

### Design

A randomized, double-blind, parallel-group, placebo-controlled trial was conducted at 10 institutions in Japan between April 2006 and October 2007. The study protocol was approved by the respective Institutional Review Boards, including the ethical committee of Keio University School of Medicine (Registration No. 17–2705), and the study was conducted according to the Good Clinical Practice Guidelines. All patients provided written informed consent before enlisting in the study. The 40 patients in the 1% BHB eye drop group were divided into 10 blocks (one block included four cases) and assigned a number from 1-1 to 10-4; likewise, the 40 cases in the placebo group were divided into 10 blocks. These blocks were randomly allocated by a disinterested party.

### Patients

We enrolled in the present study men or women older than twenty years who had been diagnosed with dry eye disease based on the Japanese diagnostic criteria^21^: a Schirmer score without anaesthesia of 5 mm/5 minutes, or a tear BUT of 5 seconds. The signs and symptoms of dry eye, except the Schirmer score, were evaluated at baseline on the day of enrolment. Subjects who had both fluorescein staining and subjective symptoms were enrolled. The enrolment process had two steps: (1) selection of cases that had been definitively diagnosed with dry eye in at least one eye according to the Diagnostic Criteria of the Dry Eye Society (1995) at the time of informed consent, and (2) a fluorescein corneal staining score (0 to 9) of 3 or higher in at least one eye during the two-week observation period. This process was necessary to select patients whose symptoms were not improved by placebo eye drops. Only patients over the age of 20 years were selected, because that is the age at which informed consent can be given autonomously in Japan.

### Exclusion criteria

Subjects were excluded who had Stevens-Johnson syndrome, ocular pemphigoid, anatomically or functionally abnormal eyelids, or a non-dry eye external eye disease; also excluded were those who had undergone hematopoietic stem cell allograft, chemical or thermal cauterization of the corneoconjunctiva, intraocular surgery during the three months prior to baseline, or lacrimal punctal occlusion. In addition, we excluded subjects who had used other dry eye medications during the study, who required contact lenses, who had participated in other clinical trials during the previous four months, or who were, may have been, or wanted to become pregnant, or were lactating.

### Study protocol

The study comprised a two-week run-in phase followed by a four-week treatment phase.

### Study medication

The medication was packaged in plastic unit-dose vials and provided as a sterile solution of 1% weight/volume BHB, or a placebo (phosphate buffered saline).

### Study treatment

During the run-in period, subjects received the placebo (vehicle) eye drops for two weeks and were instructed to use them a minimum of six times daily in both eyes. Patients who successfully completed the wash-out phase were then given the assigned medication and instructed to use it six times (more than one-hour intervals) daily for four weeks in both eyes.

### Efficacy analysis

Corneal fluorescein staining was assessed at the upper, middle, and inferior areas of the cornea, and the scores obtained were summed—each area was graded on a scale from 0 (none) to 3 (severe)^28^. Similarly, corneal and conjunctival rose bengal staining was measured in the nasal conjunctiva, temporal conjunctiva, and cornea—each was graded on a scale from 0 (non) to 3 (severe)^29^. The tear film BUT was measured three times, and the mean value was calculated^30^. The Schirmer test was performed using a standard Schirmer strip, which was kept in the lower conjunctival sac for five minutes. Symptoms of ocular discomfort, such as eye strain, dryness, and foreign body sensation, were graded on a scale from 0 (do not have this symptom) to 5 (beyond endurance). Corneal and conjunctival staining were evaluated at week two and week four; as was BUT. Symptoms of ocular discomfort, as well as the Schirmer score, were evaluated at week four.

### Subgroup analysis

Subgroups were created using the Schirmer score (≤5 mm) and BUT (≤5 seconds). Among these two groups, further subgroups were made using the subjective dryness score (≥2), foreign body sensation score (≥2), and eye strain score (≥2).

### Safety analysis

Safety was assessed using an adverse event report, blood and urea laboratory testing, vital signs, visual acuity, intraocular pressure, fundoscopy, and external eye slit lamp examination. A time schedule and detailed description of each safety test is described in the protocol.

### Statistical Analysis

A power calculation was performed, using computer simulations, on the primary outcome: the difference between the 1% BHB and placebo groups in terms of the change from baseline in fluorescence staining score. Assuming a standard deviation of 2.56 for the change in fluorescence staining score from baseline to week four, and a 20% dropout rate, it was calculated that 80 subjects (40 per treatment group) were required to achieve a ≥80% power to detect a difference of 1.84 between the 1% BHB and placebo groups in terms of fluorescence staining score. This calculation was based on using a two-sample *t*-test, with a two-sided significance level of p < 0.05.

In cases where data were available from both eyes, only the higher values obtained during the baseline fluorescein staining were used.

The Mann–Whitney U test was used to compare corneal fluorescein staining, corneal and conjunctival rose bengal staining, the subjective symptoms of ocular discomfort, and subgroup analysis between the placebo and 1% BHB groups (Fig. 6). The Student’s *t*-test was used to compare the Schirmer score and tear film BUT between the placebo and 1% BHB groups.

## Additional Information

**How to cite this article**: Kawakita, T. *et al*. Randomized, Multicenter, Double-Blind Study of the Safety and Efficacy of 1%D-3-Hydroxybutyrate eye drops for Dry Eye Disease. *Sci. Rep.* **6**, 20855; doi: 10.1038/srep20855 (2016).

## Figures and Tables

**Table 1:** Patient enrollment, demographics and adverse events.

	1% BHB	Placebo
Age	59.7 ± 15.3	59.0 ± 14.4
Gender		
Male	1	1
Female	31	32
Enrolled	32	33
Complete	26	31
Incomplete	6	2
Protocol violations	4	1
Discontinued	0	1
Dropped out	2	0
Adverse events		
Eye sensation	2 (6.3%)	0
Eye pain	1 (3.1%)	0
Eye itching	0	2 (6.1%)
Corneal erosion	1 (3.1%)	0
Conjunctival hypermia	3 (9.4%)	0
Occult blood (urine)	0	1 (3.0%)
